# RNA directed DNA methylation and seed plant genome evolution

**DOI:** 10.1007/s00299-020-02558-4

**Published:** 2020-06-27

**Authors:** R. Wambui Mbichi, Qing-Feng Wang, Tao Wan

**Affiliations:** 1 Key Laboratory of Plant Germplasm Enhancement and Specialty Agriculture, Wuhan Botanical Garden, Chinese Academy of Science, Wuhan, Hubei 430074 China; 2 Key laboratory of Southern Subtropical Plant Diversity, Fairy Lake Botanical Garden, Shenzhen & Chinese Academy of Science, Shenzhen, China; 3 University of Chinese Academy of Sciences, Beijing, 100049 China; 4 Sino-Africa Joint Research Center, Chinese Academy of Science, Wuhan, 430074 China; 5 Center of Conservation Biology, Core Botanical Garden, Chinese Academy of Sciences, Wuhan, 430074 China

**Keywords:** RNA directed DNA methylation, Seed plants, Genome evolution, Transposon regulation, Development

## Abstract

RNA Directed DNA Methylation (RdDM) is a pathway that mediates de novo DNA methylation, an evolutionary conserved chemical modification of cytosine bases, which exists in living organisms and utilizes small interfering RNA. Plants utilize DNA methylation for transposable element (TE) repression, regulation of gene expression and developmental regulation. TE activity strongly influences genome size and evolution, therefore making DNA methylation a key component in understanding divergence in genome evolution among seed plants. Multiple proteins that have extensively been studied in model plant *Arabidopsis thaliana* catalyze RNA dependent DNA Methylation pathway along with small interfering RNA. Several developmental functions have also been attributed to DNA methylation. This review will highlight aspects of RdDM pathway dynamics, evolution and functions in seed plants with focus on recent findings on conserved and non-conserved attributes between angiosperms and gymnosperms to potentially explain how methylation has impacted variations in evolutionary and developmental complexity among them and advance current understanding of this crucial epigenetic pathway.

## Introduction

**Fig. 1:**
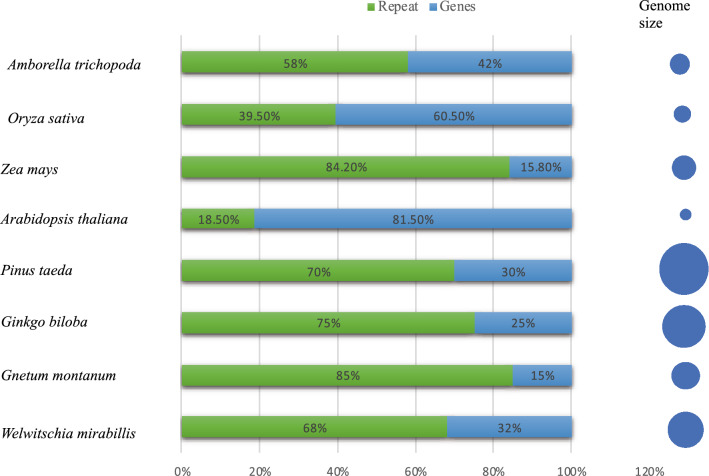
The co-relation between DNA methylation, genome size and level of Repetitive elements in sample seed plants. Species with a larger genome sizes display high percentage of repetitive elements

DNA methylation refers to addition of a methyl group (CH_3_) to the 5′ position of a cytosine base resulting in changes to activity of a DNA segment without changing its sequence (Tammen et al. 2012). Methylation occurs predominantly in TE and repeat element regions and functions to repress their activity. DNA Methylation evolved early among land plants and differs among various lineages (Leitch 2012). It has been hypothesized to have evolved primarily for control of repetitive elements within genomes, with additional functions such as regulation of gene expression and developmental functions evolving later (Bräutigam and Cronk 2018). Genomes of nearly all organisms contain repetitive elements and transposons which are mobile elements that move via cut and paste or copy and paste mechanisms (Fedoroff 2012). Consequently, plant species whose genomes have high proportions of TEs such as *Zea mays* and conifer *Picea abies*, (84.2%), (70%) respectively are very densely methylated (Ausin et al. 2016) (Fig. 1).

Repetitive element recombination activities such as insertion and deletion strongly impact the structure and evolution of genomes, as TEs accumulate and cause mutations throughout genomes (Ito 2011; Baidouri and Panaud 2013) resulting in transposon mediated genomic changes, attributable to the *C*-value paradox (Lee and Kim 2014). Angiosperm and gymnosperm genomes differ in size and structure as well as in levels and contexts of methylation. For instance, angiosperm genomes vary 2400-fold between *C*-values of lean *A. thaliana* and giant fritillaria with most species tending towards smaller *C*-values*.* On the contrary, gymnosperm genomes lack wide variation with only 16-fold *C*-value variation but lean towards larger sizes (Leitch 2012; Pellicer et al. 2018).

Apart from epigenetic silencing via methylation, plants also utilize illegitimate and unequal homologous recombination to excise TEs and counteract their duplication and recombination activities, yielding truncated and solo LTRs (Ma et al. 2004). Unequal intrastrand recombination between homologous LTR elements results in net loss of DNA by removing a portion of individual LTR and leaving a solo LTR (Bennetzen 1997; Devos et al. 2002). On the other hand, illegitimate recombination involves intra-element recombination and gradually eliminates LTRs by accumulation of small deletions in the genome and net DNA loss. The ratio of solo to intact LTRs would reflect the rate of TE removal. Genome size is therefore a factor of efficacy of TE removal mechanisms and rate of TE accumulation which varies among species (Cossu et al. 2017). For example, fritillaria have the largest angiosperm genomes ranging 30.15–85.38 Gb, resulting from accumulation of TEs due to lack of proper removal processes (Kelly et al. 2015). Interestingly, fritillaria was also found to be lacking SAWADEE HOMEODOMAIN HOMOLOGUE 1 (SHH1) and KOW DOMAIN CONTAINING TRANSCRIPTION FACTOR 1 (KTF1) genes involved in Polymerase IV&V functioning respectively in the RdDM pathway (Ma et al. 2015) similar to gymnosperms possibly a characteristic of larger genomes. Likewise, *Z. mays* genome which is relatively large (2.3 Gb) compared to its relatives has undergone TE amplification over the past three million years (Schnable et al. 2014) and has a slow rate of TE excision with a solo to intact LTR ratio of 0.2:1. On the contrary, *Oryza sativa* genome has remained small (0.5 Gb) in spite of accumulation of TEs over a similar period to *Z. mays* potentially due to effective removal of TEs with a solo to intact LTR ratio of 1.5:1 (Ma et al. 2004). Notably, among gymnosperms it was previously thought that their large genomes are due to poor TE removal mechanisms similar to observations in conifer *P. abies* which displays a large genome (19.6 Gb) as a result of a slow accumulation of TEs over millions of years and poor removal mechanisms with a 0.16:1 solo to intact LTR ratio (Nystedt et al. 2013). This has been disputed in (Wan et al. 2018) where measurement of unequal and illegitimate recombination in *Gnetum montanum* (4.1 Gb) revealed a significantly high solo to intact LTR ratio; 2.35:1 indicating more efficient TE removal and no recent TE activity, a trend that is consistent in *Amborella trichopoda* another ancient genome. These findings dispute the generalization of similarities among large gymnosperm genomes and point to conifer potentially being unique. However, an anticorrelation is theorized to exist between recombination based elimination of TEs and heterochromatization driven by epigenetic mechanisms as observed in *P. abies* and fritillaria*.* (Cossu et al. 2017) All together, these examples point to inefficiency of TE removal mechanisms as an important contributing factor to expansion in genome size among seed plants.

**Fig. 2:**
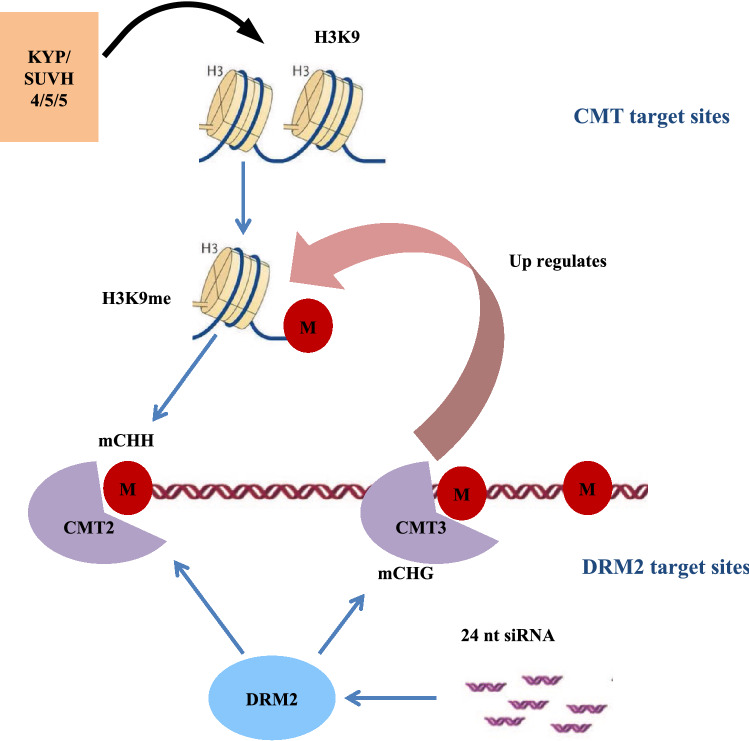
Establishment and maintenance of non-CG methylation occurs as a result of partially overlapping pathways that utilize DRM2 and CMT genes. CHG methylation is maintained by a cross talk between CMT3 and H3K9me demethylation while CHH methylation is maintained by continuous de-novo methylation involving DRM2 (marked as DRM2 region) and 24nt siRNA and also by CMT2 (marked as CMT2 region)

Methylation occurs in different cytosine contexts symmetrical CG and CHG (H = A, T or C) and asymmetrical CHH (Law and Jacobsen 2010); established by DOMAINS REARRANGED METHYLTRANSFERASE 2 (RDM2). METHYLTRANSFERASE 1 (MET1) maintains CG methylation while CHG methylation is maintained by a plant specific CHROMOMETHYLASE 3 (CMT3) (Zhang et al. 2018). Non-symmetrical CHH methylation is maintained in part by CHROMOMETHYLASE 2 (CMT2) and via constant de-novo methylation by DRM2 (Shen et al. 2014) (Fig. 2). CG, CHG and CHH methylation vary among seed plants.

This review will compare and contrast recent findings and current understanding of aspects of evolution, occurrence, regulation, and functions of methylation in angiosperms and gymnosperms and show their contribution to their divergent genomes and structures. It must be noted that angiosperms have several well-characterized methylomes unlike gymnosperms; which due to their large genome sizes comprising largely of TEs and repeats are less studied. The only gymnosperm high resolution single base resolution methylome available is that of *P. abies* (Ausin et al. 2016). We propose that continued divergence of methylation patterns has resulted in current different developmental and genomic complexity existing among seed plant species. To illustrate this, the review will cover RdDM dynamics and currently known functions in varying angiosperm species in comparison with the available gymnosperm information which is mostly limited to conifer *P. abies* and predict how these aspects of methylation have influenced seed plant genomes.

## Establishment of methylation

### Canonical pathway

Using ChIP-seq, genes involved in the RdDM pathway have been identified in model plant *A. thaliana* (Zhang et al. 2006) as well as model crop plants such as *O. sativa* and *Z. mays*. RdDM occurs in a canonical and non-canonical format each distinguished by the proteins involved, with their occurrence similarly varying among species (Matzke et al. 2015). First siRNA are generated in a process directed by RNA Polymerase IV (Pol IV), which is recruited to its target site by interacting protein (SHH1) (Law and Jacobsen 2010) which binds to histone H3K9me and unmethylated H3K4 and transcribes single stranded RNA at the target (Matzke and Mosher 2014). RDR2 associates with Pol IV and copies the single stranded RNA into double stranded RNA. DICER 3 (DCL3) then cleaves the double stranded RNA into 24 nucleotide siRNA which are made stable by HUA ENHANCER 1 (HEN1) (Haag et al. 2012; Matzke and Mosher 2014). SUVH2 and SUVH9 are SET and RING-ASSOCIATED (SRA) domain proteins and members of the SU (VAR)3-9 histone methyltransferase family involved in recruiting Pol V via their SRA domains which bind methylated DNA (Johnson et al. 2014).

**Fig. 3:**
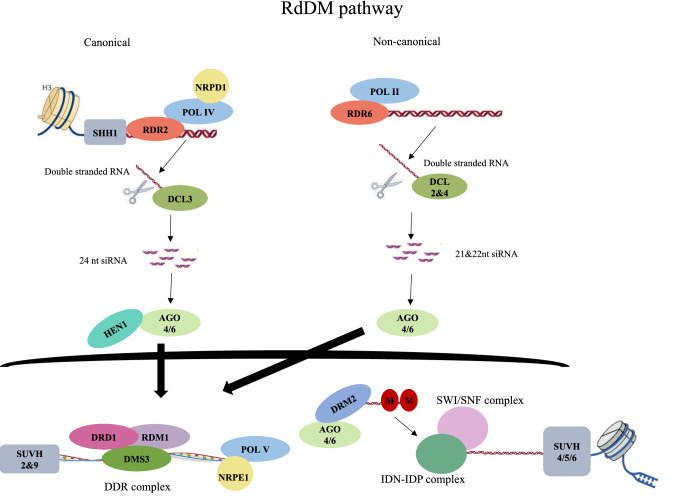
Distinction between canonical and non-canonical RdDM pathways. The key differences are illustrated i.e. the key genes involved; pol IV and DCL 3, yielding 24 nt siRNA for canonical RdDM and pol II and DCL 2 & 4 yielding 21&22 nt siRNA for non-canonical RdDM

Upon recruitment, Pol V associates with chromatin facilitated by a chromatin remodeling (DDR complex) which comprises of DEFECTIVE IN RNA DIRECTED DNA METHYLATION 1(DRD1), DEFECTIVE IN MERISTEM SILENCING 3 (DMS3) and RNA DIRECTED DNA METHYLATION 1 (RDM1) (Matzke and Mosher 2014) Pol V recruits AGO4 via its largest subunit NRPE1 that contains an AGO-hook. The siRNA bound to AGO4 base pairs with Pol V transcript recruiting DOMAINS REARRANGED METHYLTRANSFERASE 2 (DRM2) to catalyze de-novo methylation (Matzke and Mosher 2014; Zhang et al. 2018). SWI2/SNF2 complex is an ATP-dependent chromatin remodeler complex which interacts with INVOLVED IN DE-NOVO METHTLATION (IDN2) and IDN2 paralogues (IDP1 & IDP2) IDN2-IDP complex and bind dsRNA to facilitate RdDM (Ausin et al. 2012). At this stage, removal of active histone marks i.e. histone de-acetylation, de-methylation and de-ubiquitination occurs followed by deposition of repressive histone modification H3K9me by SUVH4, SUVH5 and SUVH6 at some RdDM targets resulting in stable heterochromatin (Fig. 3) (Matzke and Mosher 2014; Gentry and Hennig 2014).

Previously, Pol V methylation was hypothesized to have evolved to facilitate the diploidization of polyploids and that it’s activity was stimulated by genome doubling shock so as to target duplicated genes and transposons (Craig et al. 2014). This hypothesis is likely to be supported by the occurrence of NRPE1 the largest subunit of Pol V in conifer *P. abies* and other gymnosperms but warrants further comment.

### Non-canonical pathway

The canonical pathway has been extensively studied in plants. However, several pathways of RdDM have emerged different from the canonical RdDM pathway which was discovered in relation to silencing virus infections. These pathways utilize DCL2, RDR2, AGO4, Pol IV & Pol V and 21–22 nt siRNA (Marí-ordóñez et al. 2013). Gymnosperms *P. abies*, *Ginkgo biloba* and *Welwitschia mirabilis* predominantly display 21 nt siRNA (Ma et al. 2015) making this a key difference in RdDM between angiosperms and gymnosperms. Factors involved in non-canonical methylation are typical in Transcriptional and Post-transcriptional gene silencing (TGS & PTGS).

Previous studies hypothesized that the canonical RdDM pathway is unique to angiosperms (Ma et al. 2015) however, this hypothesis has been disproved (Ausin et al. 2016) due to findings that several key genes involved in both pathways are also conserved in gymnosperms. Non-canonical RdDM mechanisms have been implicated in initiation of TE silencing via virus induced gene silencing (Bond and Baulcombe 2014) since Pol IV and Pol V are recruited by preexisting heterochromatin marks. It is noteworthy that different mechanisms have varying entry points into the non-canonical pathway and play minor roles compared to the canonical RdDM. Future studies should focus on understanding how plants transition between the canonical and non-canonical pathways and whether the two have different functions (Fig. 3).

## Maintenance of methylation

CG methylation is present both in heterochromatin regions as well as in genic regions, contrary to CHG and CHH methylation, which are predominantly present in heterochromatic regions. Different pathways regulate maintenance of each sequence context. CG methylation is maintained by (MET1), maintenance of CHG methylation involves crosstalk between CHROMOMETHYLASE 3 (CMT3) and H3K9 histone methyltransferase for demethylation (Law and Jacobsen 2010). RdDM has also been found to contribute to restoration of symmetrical methylation as observed in *A. thaliana* centromeric repeats, in a process mediated by siRNA (Teixeira et al. 2009).

On the other hand, asymmetrical CHH methylation is maintained by continuous de-novo methylation catalyzed by RDR2 and siRNA via continuous RdDM and by CMT2 to an extent (Law and Jacobsen 2010; Stroud et al. 2013). DRM2 target sites are evolutionarily young, short edges of TEs whereas CMT2 targets long TEs with high H3K9 methylation. DECREASED DNA METYLATION 1 (DDM1) is a nucleosome remodeler preferentially involved in methylation of heterochromatic sequences by counteracting the linker histone HI and CMT2 in an RdDM independent way (Zemach et al. 2013). However, no specific differences have been observed between the two in methylation context percentage.

### Gene body methylation (GbM) and Non-CG methylation

Various species carry gene body methylation (GbM) in CG context and much less in non-CG context apart from gymnosperm *P. abies* (Ausin et al. 2016) which was found to be higher than other plant species such as *A. thaliana* and *O. sativa* in the CHG context in the gene body, potentially due to its large genome size with higher proportion of repetitive elements. GbM is located in long exons and commonly occurs in constitutively expressed genes i.e. house-keeping genes and less frequently in genes of variable expression in a conserved manner across land plants (Zilberman 2017). It is interesting to note that GbM has been conserved in eukaryotes for millions of years and varies significantly across land plant species (Takuno et al. 2016). The debate on gene body methylation functionality in shaping genomes is ongoing with contradicting studies; some hypothesizing that GbM occurs as an inconsequential result of TE methylation pathways and genes and plays no role in transcription or chromatin modification (Bewick et al. 2017). This hypothesis has however been disputed in *O. sativa* and *A. thaliana* where imprinted methylation occurs in genes of maternal sex cells involved in control of transcription factors for regulation of plant development (Rodrigues and Zilberman 2015) implicating GbM in developmental processes. Among angiosperms, there is variation in GbM among various lineages (Niederhuth et al. 2016; Takuno et al. 2016) e.g. poceae have enrichment of CHH in genic regions. Such differences may be further evidence for evolutionary variations and history observed among species.

Non-CG methylation is also complex and its functions not well characterized. It requires CHROMOMETHYLASES (CMT2&3) which are plant specific methyltransferases that evolved prior to the diversification of land plants. However, CMT2 is hypothesized to have evolved with the angiosperm specific duplication (≥ 236 MYA) (Bewick et al. 2017) consistent with studies that have found CMT2 absent in gymnosperm species (Ma et al. 2015; Ausin et al. 2016).

It is noteworthy that non-CG methylation (CHG & CHH) setting and maintenance overlap and incorporate histone modifications (Stroud et al. 2013). CMTs contain 3 domains BAH, CHROMO and C-5 domains; the CHROMO domain of CMT3 binds to H3K9me while the SRA domain of histone methyltransferase SUVH binds to methylated DNA creating a self-reinforcing loop that maintains non-CG methylation. Both CMT2 and CMT3 are involved in CHG and CHH methylation though CMT3 prefers CHG sites (Fig. 2).

### DNA demethylation

DNA demethylation occurs passively after replication due to lack of a methyl donor resulting from inactivation or reduction of methylation enzymes (Li et al. 2018) or actively via demethylase enzymes that remove methylation marks (Zhang et al. 2018). Passive demethylation occurs during formation of male gametophytes and endosperm where RdDM factors were found to be reduced (Keith et al. 2009). In contrast, active demethylation utilizes DNA glycosylases DEMETER (DME) which is more specific to maternal central cell. REPRESSOR OF SILENCING 1 (ROS1) and DEMETER LIKE 2&3 (DMT2&3) act on both somatic and non-somatic tissues (Ooi and Bestor 2008) to remove methylated cytosines. Finally this is followed by a base excision repair (BER) pathway mechanism (Li et al. 2018).

Demethylases have specific target loci, ROS1 targets are established by a histone acetyltransferase INCREASED DNA DEMETHYLATION 1 (IDM1) which binds methylated DNA and acetylates histone 3 (H3) lacking H3Kme2 and H3Kme3 (Zhang et al. 2018) the mechanism is however not fully understood. ROS1 targets tend to be protein coding genes with close proximity to highly methylated TEs and is therefore thought to function in preventing spread of methylated cytosines to nearby genes (Tang et al. 2016).

Studies into regulation of demethylases have revealed a cross talk between active demethylation and RdDM. It has been hypothesized that some RdDM targets are differently regulated by ROS1 demethylase to maintain a methylation homeostasis (Lei et al. 2015). Demethylation counteracts with RdDM to prevent hyper methylation at the target loci (Gong et al. 2002; Lei et al. 2015). Further analysis in *A. thaliana* revealed that ROS1 expression was greatly reduced in mutants of RdDM factors such as NRPD1 (Tang et al. 2016) providing further proof that the two mechanisms are cross-linked. Further studies are needed to clarify the genome-wide counteraction of RdDM and active demethylation since DNA demethylation plays a role in development during embryogenesis in angiosperms and gymnosperm as seen in *P. abies*.

### Role of RdDM in transposon silencing and regulation of gene expression

Transposon silencing and regulation is the main known function of DNA methylation to date. It is an ancient function that has been identified across most land plant lineages with different methylation genes presenting varying origin times and gene structures; with genes being gained and lost within seed plant lineages particularly in angiosperms. However, RdDM gene domains are highly conserved among species pointing to the importance of their functionality (Pei et al. 2019). Transposable elements exist in multiple families and are diverse among species. They are ubiquitous in nature and may spread rapidly within genomes resulting in null and deleterious mutations within genomes. Such mutations, present a threat to genome stability and integrity which may cause plant structural and developmental defects (Deniz et al. 2019). To prevent this, DNA methylation mechanisms are triggered via various mechanisms, one of which is repeat induced mutations (Freitag and Selker 2005).

TE silencing by methylation is evidenced by the enrichment of methylation along repeat regions coupled with histone modifications and heterochromatization. This hypermethylation maintains integrity of the genomes by repressing TEs (Slotkin and Martienssen 2007). Studies conducted in *A. thaliana* and *Z. mays* indicate that mutants in RdDM factors display TE de-repression and increased transposition of TE families such as *Copia* long terminal repeat retrotransposons (LTR-RT) compared to wild types (Gaut and Hollister 2009). In angiosperms, reactivated TEs were re-silenced via RdDM while no re-activated transposons were found in gymnosperms. It is likely that gymnosperms utilize alternative transposon silencing mechanisms (Ma et al. 2015).

**Fig. 4:**
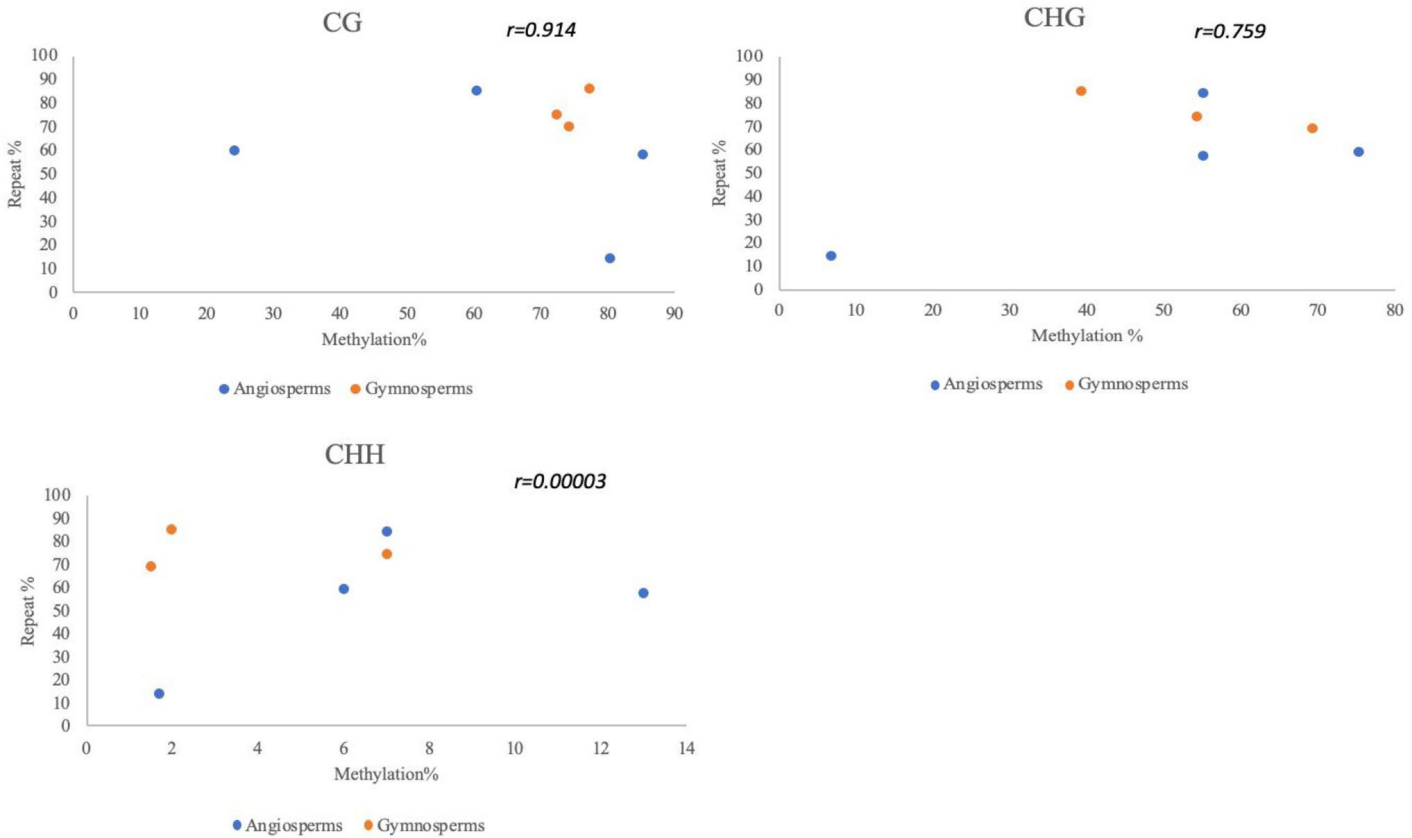
Variations in context of methylation relative to repeat percentage. **a**–**c** The scatter-plots illustrate Pearson *R* correlation coefficient between repeat content and CG *r* = 0.914 (**a**), CHG *r* = 0.759 (**b**) and CHH *r* = 0.00003 (**c**) contexts of methylation percentages in sample angiosperm and gymnosperm species

We conducted a Pearson R correlation test between repeat element and methylation percentage in different contexts using seven previously studied sample seed plant species (Fig. 4), including three gymnosperms; *G. biloba, P. taeda, G. montanum* and four angiosperms; *A. thaliana, O. sativa, Z. mays, A. trichopoda*. This revealed a strong positive correlation in CG and CHG contexts (*r* = 0.914 and *r* = 0.759) respectively while CHH methylation displayed a correlation coefficient (*r* = 0.00003) consistent with similar findings that global and gene body mCG and mCHG positively correlate with genome size (Ausin et al. 2016; Takuno et al. 2016). These findings suggest that symmetrical methylation CG and CHG also positively correlate to repeat content. Overall, genomes with high levels of TEs such as *Z. mays* (84%) and *P. abies* (70%) are densely methylated as proportion of TEs correlates positively to levels of methylation.

Comparably, DNA methylation regulates gene expression in a genome size dependent manner. Angiosperms such as *Z. mays* with larger genomes than *A. thaliana* and higher TE content undergo spreading of silencing to genes adjacent to TEs, in particular promoter regions of genes (Craig et al. 2014). Methylation in these species therefore plays a role in regulating gene expression and mutations may result in lethal abnormalities.

Gene regulation occurs through repression of transcription by preventing the binding of transcription factors or promoting repressive histone modifications such as H3K9me (Domcke et al. 2015). Alternatively, methylation activates transcription i.e. methylation at ROS1 promoter activates transcription. This function was proposed to have likely evolved secondary to TE silencing among eukaryotes and was instrumental in shaping their functioning (Bräutigam and Cronk 2018). Transcription regulation results in differential methylation among tissues and impacts physiological processes for plant growth and development during their life cycle (Zhong et al. 2013) DNA methylation gene regulation has been hypothesized to play several important roles in different tissue and cell types during developmental processes. As a whole, DNA methylation functioning is highly dependent on TE and repeat content and observing such dynamics in more gymnosperms which have even larger genomes and repeat contents will further clarify this.

## RdDM and developmental regulation

### Embryogenesis and pattern formation

**Fig. 5:**
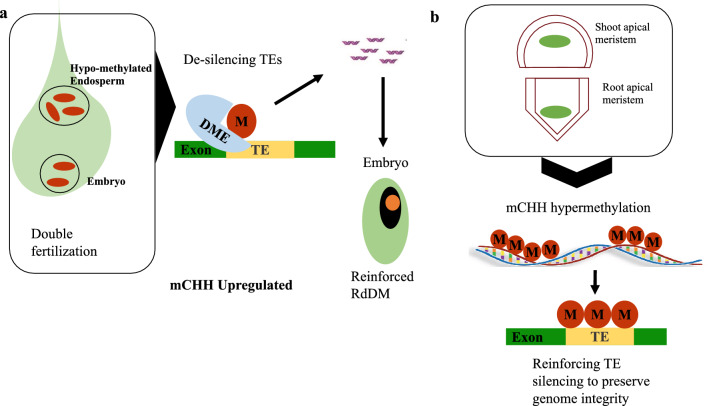
Embryogenesis and pattern formation. **a** During double fertilization demethylase DME de-silences TEs (indicated by removal of the red circle marked M) resulting in hypo-methylated gametes and endosperm. siRNAs are produced from TE transcripts and travel to the embryo to reinforce RdDM (red circle marked M) in particular CHH methylation is upregulated. **b** SAM and RAM tissues (green oval regions) display hypermethylation (red circles marked M) and reinforce silencing of TEs during cell differentiation for organogenesis

Flowering plants utilize double fertilization in the formation of endosperm and embryo (Raghavan 2003). During this process, endosperms in *O. sativa* and *A. thaliana* display global hypomethylation compared to embryos as a result of (DME) demethylation, occurring in the central cell of the female gamete and the vegetative cell of the male gamete (Park et al. 2016). The demethylated transposons result in production of siRNA which travel from the vegetative cell to the sex cell and reinforce RdDM to enhance transposon silencing after fertilization (Sroufe et al. 2012) (Fig. 5a) Interestingly, CHH methylation is upregulated during seed development and reduces later during germination via epigenetic reprogramming. A similar mechanism exists in conifer *P. abies* where methylation reprogramming was observed in culture cells (Ausin et al. 2016), suggesting that epigenetic reprogramming is a conserved phenomenon in both gymnosperms and angiosperms.

In addition, plants differ from animals as they do not define their germ cells during earlier developmental stages and therefore utilize already differentiated regions of plant growth containing stem cells referred to as meristems occurring at the roots and shoots; Root Apical Meristem and Shoot Apical Meristem. Meristems are progenitors for descendant cells and organs and are particularly vulnerable to deleterious somatic mutations that result from TE recombination activities. If inherited, such mutations would affect the integrity of progeny genomes (Gutzat et al. 2018). To protect genome stability and integrity, plants utilize RdDM to reinforce TE silencing in the stem cells.

In *A. thaliana,* both root and shoot meristems display similar patterns of upregulated RdDM factors and CHH hypermethylation (Fig. 5b). A comparison between root meristem and shoot apical meristem indicated that columella cells were the highest methylated cell type recorded in *A. thaliana* (Kawakatsu et al. 2016) while shoot apical meristem (SAM) during early vegetative growth, utilized RdDM to reinforce silencing of TEs via up-regulation of RdDM factors (Baubec et al. 2014). Besides, meristem hypermethylation has been suggested to likely operate in a similar way as companion cells i.e. vegetative cell of male and central cell of female for effective TE silencing to ensure proper tissue differentiation (Kawakatsu et al. 2016).

It is likely that meristem hypermethylation is conserved in gymnosperms as observed in Norway spruce conifer *P. abies* where culture cells displayed higher levels of CHH methylation compared to needle and flower buds (Ausin et al. 2016). Conservation of these mechanisms illustrates the importance of methylation in meristem functioning particularly for the crucial role of preventing activation of TEs to ensure proper inheritance of epigenetic marks to somatic tissue as well as progeny. Taken together these findings suggest that CHH methylation plays a key role in TE silencing during pattern formation in cells that undergo rapid differentiation and may also influence maintenance of stemness in stem cells. This however warrants further comment.

### Development of reproductive structures

**Fig. 6:**
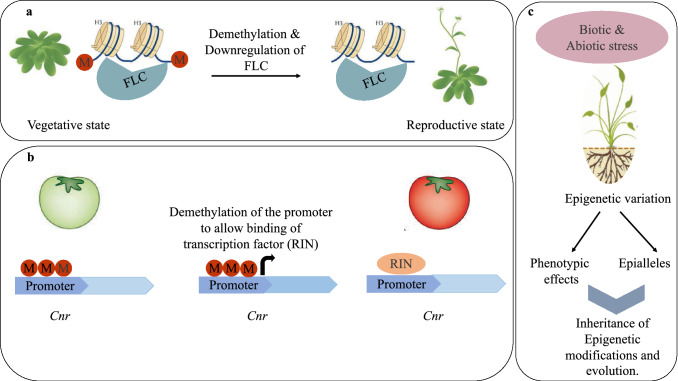
Developmental processes and response to biotic and abiotic stress conditions. **a** Vernalization during transition from vegetative to reproductive state requires the demethylation of H3 histones at FLC locus (indicated by the absence of red circles marked M) to activate its function. **b** In *Solanum lycopersicum* fruit ripening is activated by binding of transcription factor RIN to the promoter of COLOURLESS NON-RIPENING (CNR) gene locus. During the unripe state, CNR promoter is hypermethylated (indicated by red circles marked M) and must undergo demethylation to initiate the ripening process. **c** Response to biotic and abiotic stress utilizes RNA directed DNA methylation by altering epigenetic marks and creating epialleles which may be heritable to progeny as a result of epigenetic heritability

Some plants flower after extended cold periods to escape harsh winters in an epigenetically controlled phenomenon referred to as vernalization. This occurs via downregulation of protein FLC a MADS box transcriptional regulator which represses flowering (Bastow et al. 2004). In *Arabidopsis* H3 Histones within the FLC gene are methylated to repress its function and allow the transition from vegetative to reproductive state (Fig. 6a). This is an illustration of facultative epigenetic responses that allow plants to respond to environmental cues to determine developmental processes (Bräutigam and Cronk 2018). Furthermore, during fruit ripening in *Solanum lycopersicum* among other species, DNA demethylases (DML & DME) are upregulated leading to gradual demethylation at loci of ripening genes such as CNR (Zhang et al. 2018) which encodes a transcription factor CNR (colorless non-ripening). Mutants of *cnr* gene display hypermethylation at the promoter preventing binding of RIN (ripening inhibitor) (Gao et al. 2019) transcription factor and lowered gene expression (Fig. 6b). More work needs to be done to uncover additional RdDM functions in gymnosperms.

### Response to environmental factors

Plants display variations in methylation patterns upon infection by pathogens as a result of mechanisms aimed at altering susceptibility to pathogens (Yu et al. 2013). The most common example of this occurs during defense against bacterial and viral infections where DNA demethylation occurs as a part of plant induced immune response. Several plants including *Nicotiana tabacum*, *O. sativa A. thaliana* and *Cucumis sativus* have displayed an increased level of ribosomal RNA (rRNA) transcription accompanied by hypomethylation in the host plant (Yu et al. 2013; Castellano et al. 2016). Demethylation then results in recruitment of trans-activator proteins onto promoters that contain pathogen responsive elements, which regulate expression of defense genes.

Similar to biotic stress, several crop plants as well as the model *A. thaliana* have been analyzed for potential methylation roles in conditions of high ultraviolet rays, nutrient deficiency, heat (Hashida et al. 2006), cold, drought, among other abiotic stresses and illustrated variation in global or specific locus methylation (Bucher et al. 2011). These variations occurred possibly for transcriptional regulation of abiotic stress genes (Narsai et al. 2017; Li et al. 2017). In some instances these changes in the epigenome are stably inherited to the next generation (Thiebaut et al. 2019; Martienssen and Colot 2001). DNA methylation epialleles mediated by repeat sequences that cause phenotypic alterations in crop plants relay heritable changes to consecutive generations in a process thought to result from failure in epigenetic reprogramming (Fig. 6c). Studying more gymnosperm methylomes will reveal how they utilize RdDM for biotic and abiotic stress response.

## Conclusion and future outlook

DNA methylation has been shown to be a key player in maintaining genome stability and integrity among eukaryotic organisms and contributes to the diversity in genome and developmental characteristics observed among seed plant species. This review shows that methylation levels in the CG and CHG contexts positively correlate with genome size as well as repeat levels consistent with (Ausin et al. 2016). We also show that meristem functioning particularly relies on RdDM to reinforce TE silencing during differentiation in both gymnosperms and angiosperms.

Research so far has managed to illuminate majority of the key genes involved in the RdDM pathway and their roles in various stages in model plant *A. thaliana* as well as major crop plants such as *Z. mays* and *O. sativa*. Moreover, the mechanisms for maintenance and removal of methylation marks have been studied extensively. More recently, studies have revealed function of methylation in gene regulation and developmental processes. However, these revelations have also raised multiple unanswered questions such as the mechanisms by which polymerases (POL IV & POL V) and demethylases (ROS1) are recruited to their target loci. Likewise, mechanisms of non-CG methylation maintenance and establishment warrant further comment.

Further, the functions of gene body methylation and non-CG methylation remain largely unknown despite them displaying different levels in various tissue types and species (Hisataka et al. 2012; Kawakatsu et al. 2016). Notably, several growth and developmental, stress response (Hashida et al. 2006; Bucher et al. 2011), and molecular genome regulation roles that are regulated by DNA methylation have been discovered. Future research will no doubt uncover additional roles for the pathway.

**Fig. 7:**
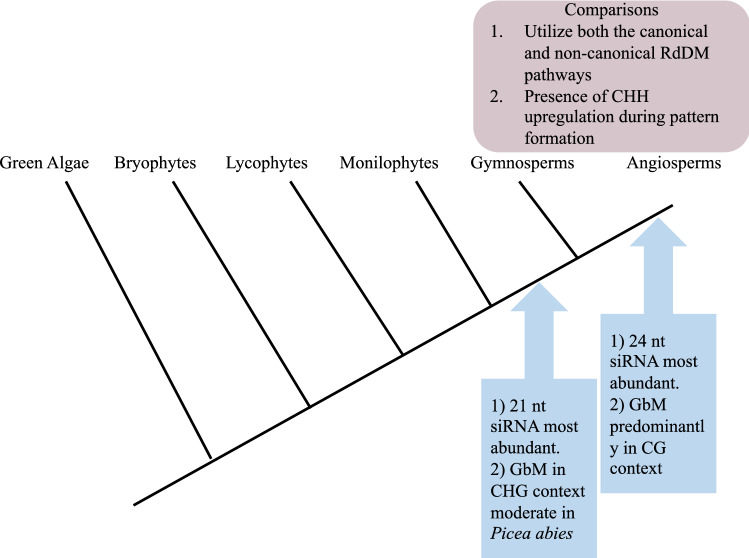
Summary of similarities and differences between aspects of RdDM gymnosperm and angiosperm

More studies need to focus on expanding to sequencing methylomes of less studied seed plant species with larger, more complex genomes as well as plants from more clades of land plants for comparison with currently available information. Such research could reveal crucial details about variations in genome evolution dynamics and more DNA methylation functions particularly in relation to seed plants (Fig. 7).

## Author contributions statement

WMR wrote the initial draft and collected the references. TW restructured the manuscript and increased the scope. QFW contributed to subsequent revisions. All authors read and approved the manuscript.

## Abbreviations

BAH domain: Bromo Adjacent Homology domain found in proteins to facilitate protein–protein interactions and plays a role in transcriptional regulation and gene silencing
CHROMO domain: Chromatin Organization Modifier is a structural protein domain utilized in chromatin manipulation and remodeling. They are also involved in gene regulation
C5 domain: Cytosine methyltransferase catalyzes the transfer of a methyl group and is dependent on SAM S-adenosyl methionine as the methyl donor
Central cell: A binucleate cell that comprises the female gametophyte and is directly involved in double fertilization in flowering plants to give rise to the endosperm and embryo
C-value paradox: Phenomenon in which organisms of similar complexity differ in nuclear DNA content in a haploid genome (1*c* value)
ChIP seq: Chromatin Immunoprecipitation Sequencing used to study the interaction of proteins with DNA. It utilizes chromatin immunoprecipitation and DNA sequencing
Epialleles: Genes that differ in their extent of methylation
Histone de-acetylation: Removal of acetyl groups from a histone to allow histone proteins to wrap around DNA more tightly and prevent transcription and gene expression
Histone de-ubiquitination: Removal of ubiquitin from the core histone it modifies for transcriptional regulation and chromatin modification
Illegitimate recombination: A mechanism that gradually eliminates LTRs, resulting in a net loss of DNA
KTF1: KOW DOMAIN CONTAINING TRANSCRIPTION FACTOR 1. RdDM protein involved in Polymerase V transcription for association with ARGANAUTE
LTR-RT: Long Terminal Repeat Retrotransposon are identical DNA sequences that repeat multiple times at either end of retrotransposons and are formed by reverse transcription of RNA
MADS Box: A conserved sequence motif in genes that binds DNA sequences. It often is a transcription factor involved in plant development processes
Meristem: Plant tissues that contain undifferentiated cells that undergo rapid differentiation giving rise to plant organs. They are found at the growing tips of roots and shoots
Pearson *R* correlation: Statistical analysis to measure the linear correlation between two variables indicated by a coefficient (*r*) which ranges from − 1 to 1
SHH1: SAWADEE HOMEODOMAIN HOMOLOGUE 1. Involved in recruitment of Polymerase IV by binding to methylated H3K9
Transcriptional and post transcriptional gene silencing: Mechanisms to repress transcription of genes or mRNA degradation after transcription, resulting in their silencing through DNA methylation
Trans-activators: Protein molecules that trigger activation of a gene by another molecule. It commonly occurs following infection by a virus
Unequal recombination: A type or genetic recombination by which transposable elements are excised from the genome. It leads to the elimination of two long terminal repeats and the internal region of the element leaving a Solo long terminal repeat
Vegetative cell: A component of the pollen grain that initiates formation of the pollen tube which carries the sperm cells to the ovule for fertilization
Vernalization: A mechanism in which plants use prolonged cold periods to initialize flowering allowing them to adapt to seasonal changes during development
